# Electro-Mechanical Impedance (EMI) Based Interlayer Slide Detection Using Piezoceramic Smart Aggregates—A Feasibility Study

**DOI:** 10.3390/s18103524

**Published:** 2018-10-18

**Authors:** Jianchao Wu, Weijie Li, Qian Feng

**Affiliations:** 1 Hubei Key Laboratory of Earthquake Early Warning, Institute of Seismology, China Earthquake Administration, Wuhan 430071, China; wujianchao@eqhb.gov.cn; 2 Institute of Geophysics, China Earthquake Administration, Beijing 100081, China; 3 School of Civil and Environmental Engineering, Harbin Institute of Technology, Shenzhen 518055, China; wli27@uh.edu

**Keywords:** electro-mechanical impedance (EMI), interlayer slide, piezoceramic transducer, smart aggregates

## Abstract

Interlayer slide damage is one of the main causes of landslide hazard, inflicting huge economic losses and casualties. It is urgent to accurately detect the initiation and development of the interlayer slide damage in real time. In this paper, a study on the feasibility of using the electro-mechanical impedance (EMI) technique to detect the interlayer slide damage was presented. The main purpose of this paper is to investigate the application of the EMI technique for interlayer slide detection using piezoceramic smart aggregates (SAs). In the experimental study, three small landslide specimens with a weak interlayer in the middle were fabricated. For each specimen, three piezoceramic SAs were post-embedded at specific positions, which were located above the weak interlayer inside the structure. The specimens were subjected to a compressive test to initiate an interlayer slide along the weak layer. The whole loading process was monitored with a precision impedance analyzer by measuring the admittance (reciprocal of impedance) of the SAs over time. The statistic metrics, including root mean square deviation (RMSD) and mean absolute percentage deviation (MAPD), were introduced to quantify the variations in admittance signatures due to interlayer slide damage. It was found that the admittance signatures and statistic metrics were sensitive to the interlayer slide damage. The experimental results verify the feasibility and practicality of using EMI technique to detect the interlayer slide.

## 1. Introduction

Landslides are one of the most dangerous natural disasters on the earth. Landslide usually occurs within a very short span of time, so it can result in enormous economic losses and casualties. The causes of landslide hazard are mainly attributed to the weak interlayer inside of the landslide body, which has loose structure, poor behavior, and low strength. Intense rainstorm or strong vibration could cause a rapid increase in shear stress or decrease in shear strength of the inherent weak interlayer in rock or soil [1]. Then, landslide body slides frequently along the weak interlayer surface. Therefore, landslide monitoring, especially the interlayer slide detection over time and early warning in time, is particularly of great importance to minimize property damage and losses of human lives.

To monitor the interfacial bonding condition between the weak interlayer and the surrounding medium, various techniques have been undertaken in engineering practice for decades, such as photogrammetric techniques [2,3], ground-based geodetic techniques [4,5], and satellite-based geodetic techniques [6]. Although these technologies have been applied for many years in engineering practice, they still have some shortcomings. Most of them require sophisticated and expensive equipment with experienced experts [7,8]. In addition, the overall costs for long-term and continuous monitoring are usually huge. For the photogrammetric technique, it is not sensitive to small-scale landslides and has limitations in case of extreme weather, such as heavy rainfall and dense fog weather. For the ground-based geodetic technique, it is mainly applied for relative deformation measurements. The on-site measuring instruments require regular maintenance. The satellite-based geodetic technique usually requires a line-of-sight from the monitoring receivers to the transmitting satellites, and has limitations in forested areas. To overcome these problems, a low-cost, robust, and real-time technique should be developed for landslide monitoring.

The structural health monitoring (SHM) technique is receiving increasing attention in civil and mechanical engineering. SHM has been successfully applied to the structural interface damage detection in recent decades. Moreover, the electro-mechanical impedance (EMI)-based SHM technology has been researched in a wide variety of structures [9,10,11,12,13,14,15,16]. A key point of EMI technique is the utilization of Lead Zirconate Titanate (PZT) patches as collocated actuators and sensors. The PZT-based smart aggregates (SAs), fabricated by sandwiching two PZT patches between two marble blocks, are widely researched in the SHM field [17,18,19,20,21,22,23,24]. The SAs can be conveniently embedded inside a structure and adopted in the EMI technique. The basis of this technique is the energy transfer between the transducer and its host structure. In general, this technique takes advantage of piezoceramic transducers embedded inside the structure to be measured. The transducers’ electrical impedance is able to reflect the mechanical impedance of the structure. With the advantages of low-cost, small size, sensitivity to stiffness or mass variations of the host structure, the EMI-based approach can be utilized to assess the health state of the structure. For SHM applications, the usual practice is to compare the high frequency response spectra of the electrical impedance in the initial state with damaged state. With this, most of the researches monitored the variations in the frequency response of the electrical impedance of the PZT due to changes in the mechanical impedance of the host structure. Examples of EMI-based method include stress monitoring [25,26,27], bond-slip detection [10,19], crack monitoring [11,13,28,29,30,31,32,33], corrosion assessment [34,35], and strength gain of concrete [36,37,38]. The application of the EMI technique in different areas has shown promising results up to date. It has been well established that the structural damages will cause frequency and amplitude shifts in the electrical impedance response spectra. Combined with the root mean square deviation (RMSD) and mean absolute percentage deviation (MAPD)-based damage index, the location and severity of damage can also be determined quantitatively.

Although the EMI-based technique was successfully applied in SHM of civil, mechanical, and aerospace engineering structures, its application in the geological field is rather limited. The objective of this study is to investigate the feasibility of the EMI technique for monitoring the interlayer slide by employing the SA transducers. In this study, the PZT-based smart aggregates were post-embedded into the landslide specimens. There are a total of three specimens and nine embedded SAs were utilized for detection of the electrical impedance. In addition, two damage indexes, including the RMSD and MAPD, were employed to quantitatively evaluate the occurrence and development of the interlayer slide damage. Experimental results verified the feasibility and effectiveness of the EMI technique with the help of embedded SAs for interlayer slide detection.

## 2. Smart Aggregate and Electro-Mechanical Impedance Method

### 2.1. Smart Aggregate

**Figure 1:**
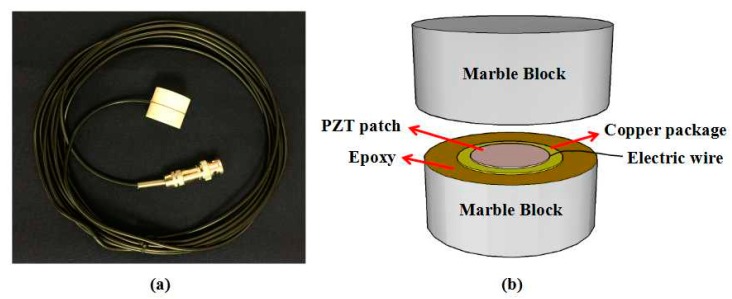
Photo of a smart aggregate (SA) (**a**) and the schematic of a smart aggregate (**b**).

The smart aggregate (SA), first proposed by Song et al. [39], is a piezoceramic-based multifunctional transducer. To protect the fragile PZT patch from external damage, the SA is fabricated by sandwiching two water-proofed PZT patches between two cylindrical marble blocks, as illustrated in Figure 1. Due to the mechanical protection, SA can provide superior embeddability and compatibility when deployed as an embedded transducer in a concrete structure. The SA has a diameter of 25 mm and a height of 20 mm. The embedded PZT patch has a diameter of 10 mm and a height of 0.3 mm. The smart aggregate has been widely used in structural health monitoring of various structures with active sensing approach [40,41,42], EMI technique [43] and acoustic emission method [44]. Previous researches have verified that the SA-based methods are feasible and effective approaches in structural health monitoring field.

### 2.2. Electro-Mechanical Impedance (EMI) Method to Detect Interlayer Slide

**Figure 2:**
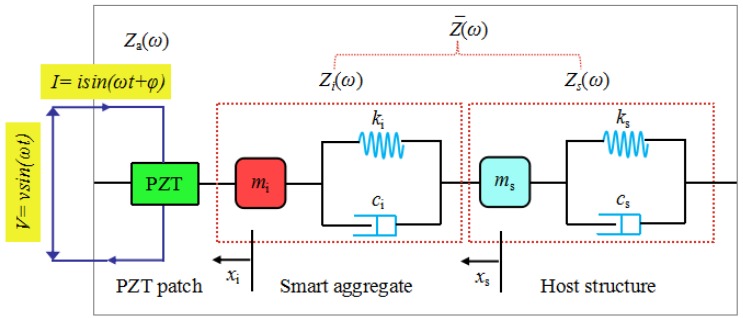
The 2D electro-mechanical impedance model of Lead Zirconate Titanate (PZT)-SA-host structure system.

For the sake of completeness, a brief description of the EMI technique is introduced here. Sun et al. first proposed the feasibility to monitor the structural health status using PZT transducers [45]. The two-degrees-of-freedom electro-mechanical impedance model of PZT interface–host structure system was proposed by Huynh and Kim and then researched by his research team [46,47,48]. This model was designed to represent coupled dynamic responses of PZT interface–host structure, as illustrated in Figure 2. In this two-degrees-of-freedom system, one refers to the host structure denoted by the impedance *Z_s_* and the other one refers to the smart aggregate denoted by the impedance *Z_i_*. The two-degrees-of-freedom spring-mass-damper system can also be adopted to demonstrate the coupling effect between a smart aggregate and the host structure. The EMI response of the two-degrees-of-freedom system *Z_ω_* was obtained by Huynh and Kim, as follows:

(1) $$Z\left(\omega \right)={\left\{i\omega \frac{w_{a}l_{a}}{t_{a}}\left[{\hat{\varepsilon }}_{33}^{T}-\frac{1}{\frac{Z_{a}\left(\omega \right)}{\bar{Z}\left(\omega \right)}+1}d_{31}^{2}{\hat{Y}}_{11}^{E}\right]\right\}}^{-1}$$

where *l_a_*, *w_a_,* and *t_a_* are the length, width, and thickness of the PZT patch, respectively. ${\hat{Y}}_{11}^{E}=\left(1+i\eta \right)Y_{11}^{E}$ is the complex Young’s modulus of the PZT patch at zero electric field; ${\hat{\varepsilon }}_{33}^{T}=\left(1-i\delta \right)\varepsilon_{33}^{T}$ is the complex dielectric constant at zero stress; $d_{31}$ is the piezoelectric coupling constant in the 1-direction at zero stress; ω is the angular frequency of the driving voltage. η is the structural damping loss factor and δ is the dielectric loss factor of piezoelectric material. The impedance is a complex value since the electric permittivity and Young’s modulus of the PZT patch are complex plural values due to the loss factors. From previous studies [45,49], it was shown that the real part of EMI contains much more information of structural integrity than the imaginary part.

As revealed in Equation (1), the two-degrees-of-freedom impedance model contains two resonant peaks in its impedance signatures, which represent two coupled vibration modes in the PZT-SA-host structure system. The impedance model represents the structural parameters of both the SA and the host structure. The interlayer bonding condition in a landslide body directly influences the mechanical impedance of the structure. The mechanical impedance of the host structure is a function of the structural mass, stiffness, and damping. Any structural change in the mechanical properties of the structure (or the SA) can cause changes in the electrical impedance of the PZT-SA-structure system. Therefore, the structural integrity of the host structure can be estimated by detecting the impedance changes. When the interlayer slide occurs, the physical and mechanical properties of the structure will be changed due to the discontinuity and interface. Therefore, the variations of the mechanical properties of the structure can be detected by measuring the changes in the EMI signatures of the PZT transducer. Analysis of the EMI signals can reveal the occurrence and development of the interlayer slide when compared with a baseline or initial condition.

## 3. Experimental Setup

### 3.1. Specimen Preparation

**Figure 3:**
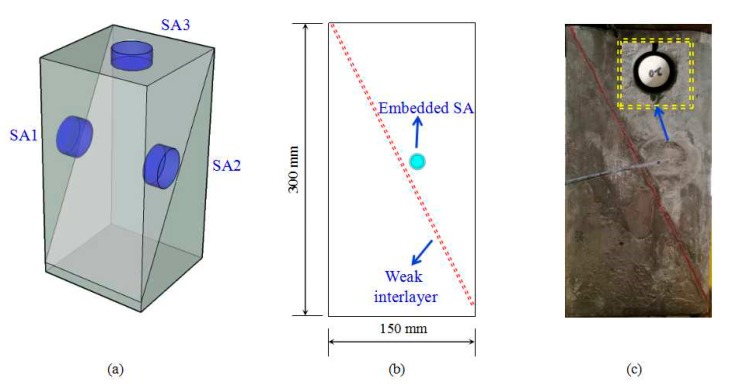
The schematic and photo of the specimen. (**a**) 3D sketch; (**b**) left lateral view; (**c**) photo.

As a highly inhomogeneous material, it is difficult to predict the exact sliding locations for the rock mass. A model test becomes a feasible and effective way to explore the rock sliding detection. To explore the feasibility and efficacy of the EMI-based technique for interlayer slide detection, a group of small landslide specimens were fabricated. Figure 3 shows the 3D schematic and photo of a specimen. The specimen has a dimension of 150 mm × 150 mm × 300 mm. The design mix of specimen corresponding to cement: sand: water by weight was 1:3:0.6. A Plexiglas plate has been proved to be appropriate and effective for the weak interlayer detection [49]. The weak interlayer in this study was simulated by a PVC Plexiglas plate, which was designed as an expected sliding plane. The dimensions of the Plexiglas plate were 280 mm, 100 mm, and 3 mm in length, width, and thickness, respectively. The Plexiglas plate was embedded in the specimen along one of its diagonal plane. It is noted that the angle between the weak interlayer and the level surface is 60°, which is consistent with the high slope in the field. All of the specimens were compacted fully using a table vibrator during the casting and demolded from the wooden frame 24 h after the casting. The specimens were then cured in the curing room for 15 days and then moved into the lab for the loading test.

In order to detect the local impedance signatures at different locations, three SAs were used for each specimen. The SAs were installed by drilling 30-mm diameter and 35-mm deep holes at different locations of the specimen, as shown in Figure 3c. The SAs were embedded and fixed inside the holes. Then, the holes were refilled by non-shrinkage mortar. After curing of the mortar, the SAs became integrated parts of the surrounding medium. As illustrated in Figure 3a, for each specimen, two SAs were embedded on the left and right lateral surface, respectively. One SA was embedded near the top surface. All of the SAs were located above the weak interlayer and the distance from the SAs to the weak interlayer was 10 mm. The groups of specimens in this study are listed in Table 1.

### 3.2. Instrumentation Setup and EMI Monitoring

**Figure 4:**
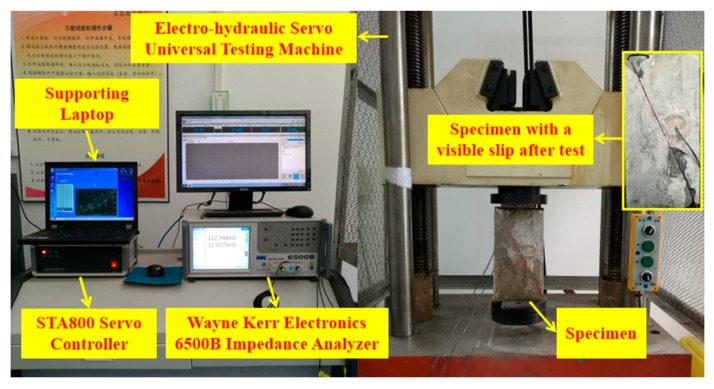
Overall experimental setup.

Experimental setup includes an impedance analyzer, a computer equipped with a data acquisition system, and a universal testing machine, as shown in Figure 4. For the experimental determination of the sliding behavior, a vertical loading test is often adopted. The vertical compression loading was achieved with a 1000 KN capacity electro-hydraulic servo universal testing machine. This machine was operated under the displacement control at a constant rate of 0.02 mm/min. The load and displacement data were registered by the electronic load cell of the testing machine.

The EMI measurement was performed by employing the Wayne Kerr Electronics 6500B impedance analyzer (Wayne Kerr Electronics, West Sussex, United Kingdom). Taking advantage of the impedance analyzer, the impedance and admittance signatures of the SAs can be directly monitored over a high frequency range. Both the real and imaginary components of the electrical impedance and admittance can be measured and demonstrated as a function of frequency. Resistance and reactance are the real and imaginary components of the impedance, respectively. While conductance and susceptance are the real and imaginary components of the admittance, which is defined as the reciprocal of impedance. In this study, only the conductance and susceptance signatures were analyzed and presented in the following analysis. The conductance and susceptance were chosen since that it has been experimentally proven to have better sensitivity in the structure’s integrity [19,50]. Baseline measurements of the SAs were recorded at a healthy stage in advance (before application of loading test). Then, the EMI signatures of the SAs were acquired and saved every 10 min using the impedance analyzer.

## 4. Experimental Results

### 4.1. Loading History

**Figure 5:**
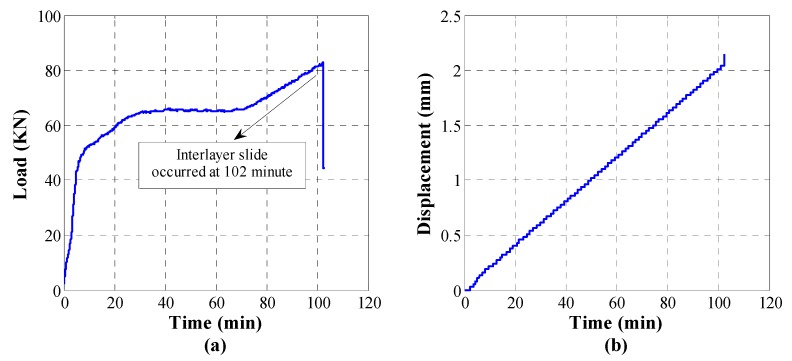
Loading history. (**a**) Load curve; (**b**) Displacement curve.

It should be noted that a total of three specimens were adopted in this study to compare and verify the results with each other. To avoid redundancy, the loading history of specimen S1 was analyzed as a representative in this paper. The entire test lasted 110 min and the loading history curve registered by the universal testing machine is shown in Figure 5. Based on the characteristics of the loading history curve, the entire sliding process can be demonstrated to a certain degree. During the early loading stage, the load–time curve shows an approximate linear relationship and no slip occurs. It revealed that elastic deformation and stress accumulation occurred inside the structure. The anti-slip strength comes from the chemical adhesion and friction mechanisms. With the gradual increase of the loading, the friction between the weak interlayer and the surrounding medium accumulated and increased. The load peaked at 102 min and dropped suddenly. The load decrease indicates the loss of the anti-slip strength and implies the initiation of the interlayer slide. When the load dropped at 102 min, a loud and crisp sound due to the slide was also heard. At the same time, a dislocation was observed along the weak interlayer on the specimen.

### 4.2. EMI Signature Measurement

**Figure 6:**
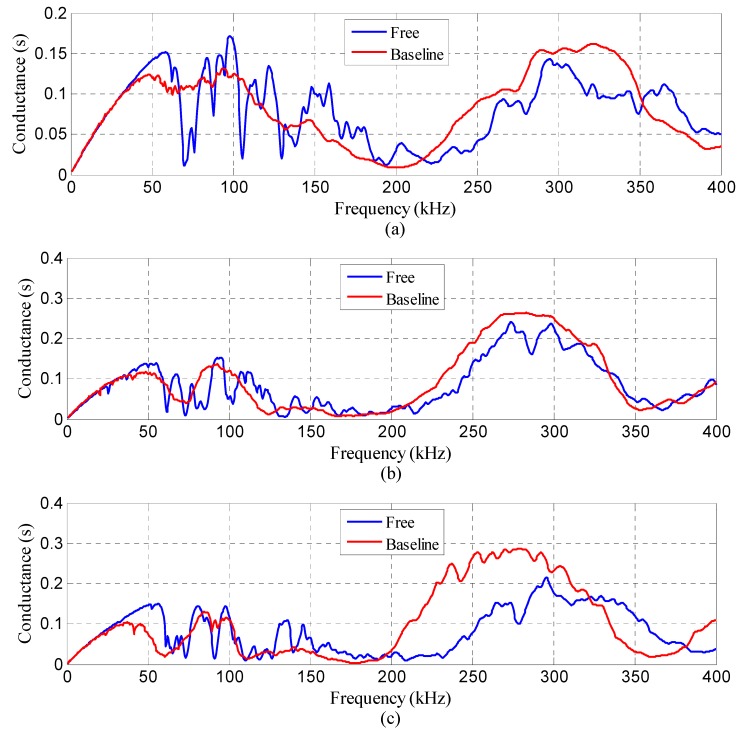
Conductance spectra between free condition and baseline condition of the three SAs in specimen S1. (**a**) SA-1, (**b**) SA-2, (**c**) SA-3.

To determine an appropriate frequency range, the conductance was first scanned over a wide frequency range prior to the test. The wide frequency range is from 1 kHz to 400 kHz in the test. Figure 6 demonstrates the conductance spectra recorded in two different conditions. As shown in Figure 6, the blue spectrum denotes the conductance signature detected when the SA was free in the air. The red one represents the result when it was embedded inside the host structure. For the purpose of high sensitivity to the incipient damage, the electrical impedance was measured at high frequencies in the range of 280 kHz–320 kHz since some significant peaks in the conductance spectra were covered. Under the frequency range, the wavelength of the excitation signal is small, which is very sensitive to minor damages and not affected by any far-field changes.

**Figure 7:**
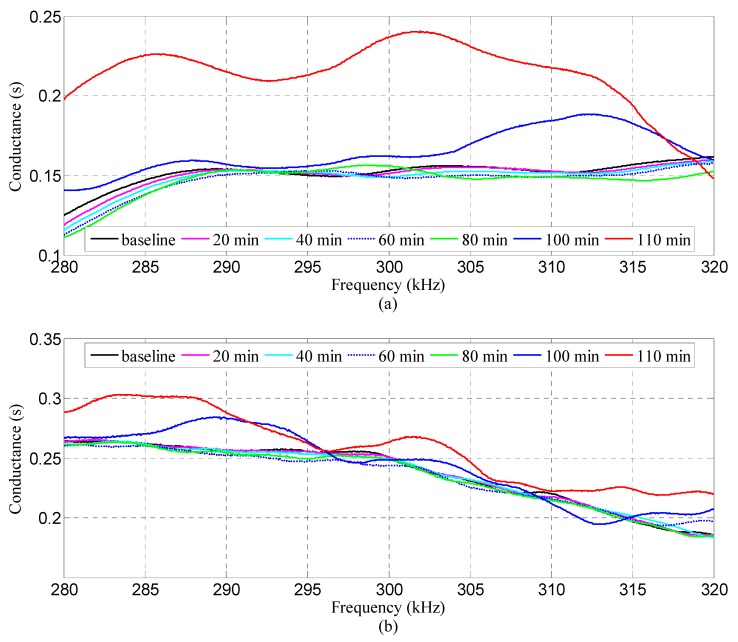

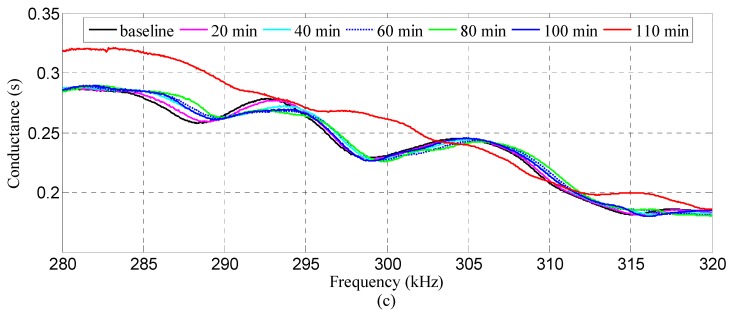
Conductance spectra of the three SAs in specimen S1. (**a**) SA-1, (**b**) SA-2, (**c**) SA-3.

Figure 7 shows the conductance spectra acquired from the three SAs inside specimen S1 during the loading test. Since the spectra are closely spaced, it is inappropriate to illustrate all the data in one figure. Only the measurements detected every 20 min are presented for further analysis, namely, those for baseline, 20, 40, 60, 80, 100, and 110 min. As shown in Figure 7, there exist small but noticeable variations for the conductance spectra at different moments as compared with the baseline measurement. Specifically, the deviation level heightens as the loading time grows. This may be explained by the fact that the internal damage along the weak interlayer became more severe. The slide that occurred at the interface between the weak layer and surrounding medium destroyed the integrity of the structure, thus inducing a variation to the local stiffness and damping. Therefore, the mechanical impedance of the structure was changed as evident with the deviations in the electrical impedance signatures of the SA transducers. It is interesting to note that the variations of the spectra from SA-3 are the smallest as compared with the results from SA-1 and SA-2. The possible reason is that SA-3 was located on the top surface of the specimen, rendering it less sensitive to the slide damage. Typically, the electrical impedance/admittance of the SA embedded in the vicinity of damage is more sensitive. Therefore, the EMI measurement registers a larger variation compared to the one located further away from the damage. The experimental results can be illustrated by the fact that the interlayer slide damage became much more severe as the friction resistance continues to increase near the weak interlayer. The stress concentration on the interface between the weak interlayer and the surrounding medium greatly affected the integrity of the original structure. Therefore, the local stiffness and damping of the structure were altered, and then the corresponding mechanical impedance was changed.

In summary, the conductance parameter seems to interpret the interlayer slide damage evolution to a certain degree. It is still qualitative analysis from the conductance spectra. With regard to this, we need to adopt quantitative assessment metrics, such as RMSD and MAPD.

### 4.3. Evaluation Using Statistics Metrics

The impedance signature plots provide a qualitative approach for slide detection in previous section. To account for the quantitative analysis in the EMI spectra during the loading process, they can be dealt with scalar damage metrics such as RMSD and MAPD. These metrics, based on frequency-by-frequency, are commonly used in structural health monitoring as damage indices. The RMSD and MAPD have been found to be effective for characterizing the initiation and evolution of the damages. The mathematical expressions of these metrics in terms of the conductance Re(*Y*) are given as follows:

(2) RMSD(%)=∑n=1N[Re(Yn,1)−Re(Yn,0)]2∑n=1N[Re(Yn,0)]2

(3) MAPD(%)=1N∑n=1N|Re(Yn,1)−Re(Yn,0)Re(Yn,0)|

where *N* indicate the number of sampling points for the impedance analyzer and *N* = 801 in this study. The subscripts 0 and 1 denote the initial measurement and subsequent measurement, respectively. The calculated results given by these metrics can quantify the difference intuitively between two or more EMI spectra at different measuring moments. For these metrics, the greater value of the metrics, the larger the difference between the initial and subsequent measurement.

**Figure 8:**
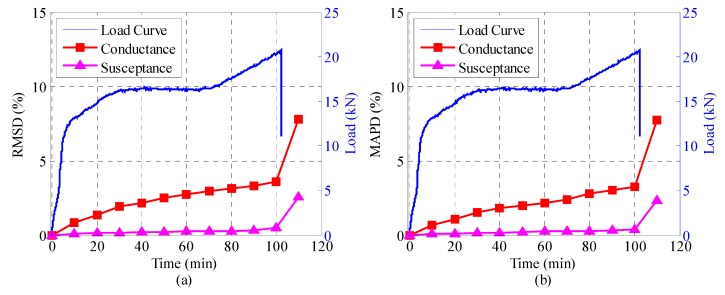
The damage metrics of the conductance and susceptance for SA-1 during the test. (**a**) root mean square deviation (RMSD), (**b**) mean absolute percentage deviation (MAPD).

**Figure 9:**
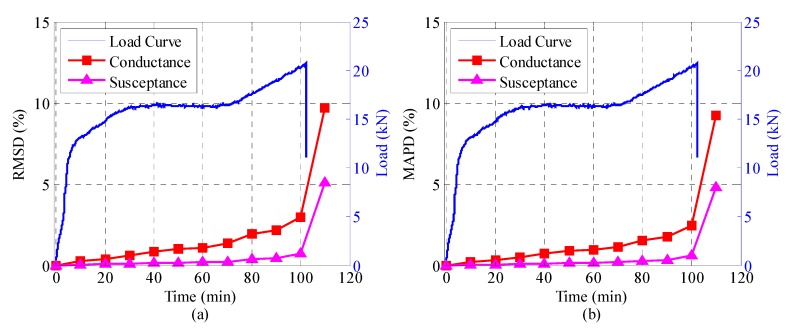
The damage metrics of the conductance and susceptance for SA-2 during the test. (**a**) RMSD, (**b**) MAPD.

**Figure 10:**
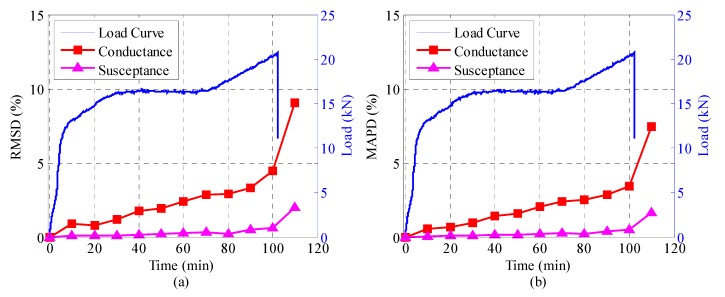
The damage matrices of the conductance and susceptance for SA-3 during the test. (**a**) RMSD, (**b**) MAPD.

Figure 8, Figure 9 and Figure 10 illustrate the RMSD and MAPD damage metrics of the admittance signatures of SA-1, SA-2, and SA-3 at different time levels, together with the load history curve. It can be seen that both the RMSD and MAPD metrics for conductance and susceptance display a resembling trend. In the first 100 min of the experiment, the metrics stay at a low value and increase slowly. The damage metrics steeply increased to a larger value at the 110th minutes, and this was the moment when the slide ends. The steeply increasing value indicates some mechanical changes occurred in the structure. The transition point can be attributed to the initiation of interlayer slide and sudden release of the friction resistance. Overall, the damage metrics including RMSD and MAPD are capable of quantifying the changes of admittance signatures due to the interlayer slide damage. They can also reflect the whole process of the interlayer slide damage. Figure 9 and Figure 10 show the results of SA-2 and SA-3, respectively. It is evident that the computed results from the two SAs are different due to the different embedded positions. However, the computed results are also sensitive to the damage. It is interesting to note that conductance have better sensitivity as compared with susceptance. As can be seen from Figure 8, Figure 9 and Figure 10, the RMSD and MAPD of conductance have larger value than that of susceptance in the entire process. Then, it is more obvious to perform damage assessment in this research. As mentioned before, conductance is the real component of the admittance and susceptance is the imaginary one. Therefore, conductance is an appropriate parameter for adopting and performing structure health monitoring.

**Figure 11:**
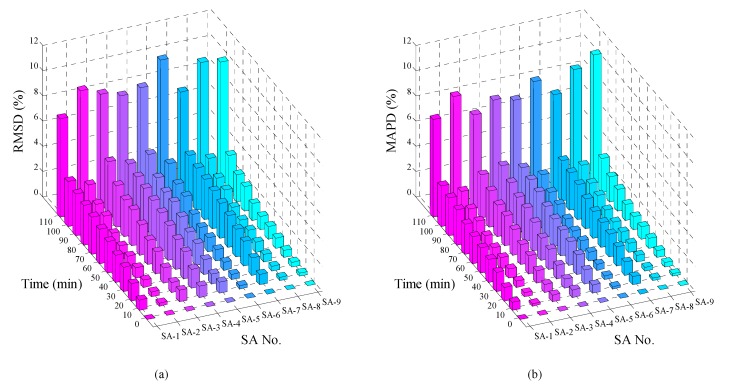
The damage matrices of the conductance for the nine SAs during the test. (**a**) RMSD, (**b**) MAPD.

As for comparison and verification, Figure 11 shows the RMSD and MAPD damage metrics of the conductance signatures of all nine SAs at different moments. It is evident that the metrics trends of the SAs are similar as compared with the previous results. As shown in Figure 11, it clearly shows the significant increases of the damage metrics for all the SAs after the slide damage. Overall, these damage metrics are able to quantify the variations of impedance signatures due to the interlayer slide. It further reveals that these metrics are able to reflect the initiation and evolution of the interlayer slide damage.

## 5. Discussions

In this study, the EMI technique for interlayer slide detection was investigated. It was verified that the proposed approach is feasible in detection of interlayer slide damage. It should be noted that the interlayer slide in rock mass cannot be simulated accurately by the loading test in laboratory. It is difficult to predict the exact sliding damage surface for the rock mass, which is a highly inhomogeneous material. It was not convincing to determine the initiation of the interlayer slide only by the measured loading curve, since the deformation under compression is complex. The loading history curve obtained in this study is closed related to the mixture ratio, interlayer angle, specimen size, and curing conditions. More experiments are needed to be performed to obtain more reliable results.

In addition, it would be a critical issue that how to differentiate the EMI signature variations of interlayer slide damage from other types of damages, such as the microcracks and the environmental conditions (temperature, moisture). In reality, there are so many factors that can cause the change of the mechanical impedance of the landslide body. So far, the structural health monitoring based on EMI technique is still concentrated in the laboratory research stage. Some researchers have discussed the practical issues of EMI in real applications in depth [51,52,53]. The issue regarding whether the variation of EMI signatures caused by specified damage or other damages has also been investigated. One method is to take advantage of the frequency dependent properties of each damage types. Different types of damage may cause the variations in the EMI signatures at different frequency ranges. To identify whether the interlayer slide or other damage cause the EMI signature variations, more investigations should be performed to study the response of the EMI signatures corresponding to each damage type, especially to identify the frequency range of different damage types. Based on the frequency dependent response of the EMI signatures to damage types, a selection of specified frequency range is able to isolate the influence of some other damage types on the EMI signatures. Another method is to incorporate other techniques, such as fiber optic sensors, guided ultrasonic waves testing, acoustic emission technique, and so on. These techniques are also widely used in structural health monitoring. Combined with the information obtained from these techniques, it is possible to characterize the characteristics of interlayer slide damage or damages from other sources. Future study should be focused on the investigation of real structures in their operational conditions.

As a new monitoring technique, there are still issues need to be addressed prior to field application. One issue related to the real field application is that the equipment is relatively bulky, expensive and not portable for field monitoring. For example, the impedance measurement in our laboratory-based study is conducted using the Wayne Kerr Electronics 6500B impedance analyzer. Consequently, the development of miniaturized, cost-effective impedance measurements system should be adopted in the real field monitoring. For a large-scale complex landslide body, the deployment of an array of smart aggregates up to hundreds is another important issue. In general, the number of sensors to be implemented is influenced by the sensing range of the smart aggregates, which is heavily dependent on the material properties and geometry of the host structure.

## 6. Conclusions

This study was conducted to investigate the feasibility and applicability of the electro-mechanical impedance (EMI) sensing technique for interlayer slide detection of a landslide body. Based on the experimental results, it was found that the piezoceramic smart aggregates (SAs) have significant potential in detecting the interlayer slide. The variations in the admittance signature over time were revealed to be an indicator to monitor the interlayer slide. The SA transducers embedded on the lateral surface responded more sensitively than the one located on the top surface. In addition, the quantitative method based on the statistics metrics, including root mean square deviation (RMSD) and mean absolute percentage deviation (MAPD), was also certified to be a reliable indicator for detecting the interlayer slide. Moreover, it has been confirmed that the real part of admittance, namely conductance, has a better sensitivity as compared with the imaginary part. Our study demonstrated that the EMI-based structural health monitoring technique can be adopted effectively to monitor the interlayer conditions of a landslide body in the near future.

## Figures and Tables

**Table 1:** The groups of specimens.

Specimen Number	Left Lateral Surface	Right Lateral Surface	Top Surface
S 1	SA-1	SA-2	SA-3
S 2	SA-4	SA-5	SA-6
S 3	SA-7	SA-8	SA-9
